# The Human Heart

**Published:** 1892-09

**Authors:** 


THE HUMAN HEART.—The workings of the human heart have been computed by a celebrated physiologist, and he has demonstrated that it is equal to the lifting of 120 tons in twenty-four hours. Presuming that the blood is thrown out of the heart at each pulsation in the proportion of sixty-nine strokes per minute, and at the assumed force of nine feet, the mileage of the blood through the body might be taken at 207 yards per minute, 7 miles per hour, 168 miles per day, 61,320 miles per year, or 5,150,880 miles in a life time of eighty-four years. In the same period of time the heart must beat 2,869,776,000 times.—*Ex.*

